# Beyond the prescription: Global observations on the social implications of GLP-1 receptor agonists for weight loss

**DOI:** 10.1371/journal.pgph.0005516

**Published:** 2025-12-26

**Authors:** Sissel Due Jensen, Bruno Gualano, Pernille Andreassen, Fernanda Baeza Scagliusi, Cindi SturtzSreetharan, Alexandra Brewis

**Affiliations:** 1 Department of Public Health, Aarhus University, Aarhus, Denmark; 2 Steno Diabetes Center Aarhus, Aarhus University Hospital, Aarhus, Denmark; 3 Center of Lifestyle Medicine, Universidade de São Paulo, São Paulo, Brazil; 4 Laboratory of Assessment and Conditioning in Rheumatology, Hospital das Clínicas, Universidade de São Paulo, São Paulo, Brazil; 5 Faculdade de Medicina, Universidade de São Paulo, São Paulo, Brazil; 6 The Danish National Center for Obesity, Aarhus University Hospital, Arhaus, Denmark; 7 Center for Lifestyle Medicine, Universidade de Sao Paulo, São Paulo, Brazil; 8 Research Group in Eating, Corporealities and Culture, Universidade de Sao Paulo, São Paulo, Brazil; 9 Department of Nutrition, School of Public Health, Universidade de São Paulo, São Paulo, Brazil; 10 Department of Clinical Medicine, School of Medicine, Universidade de São Paulo, São Paulo, Brazil; 11 Center for Global Health, Arizona State University, Tempe, Arizona, United States of America; 12 School of Human Evolution and Social Change, Arizona State University, Tempe, Arizona, United States of America; University of the Philippines Diliman, PHILIPPINES

## Abstract

Glucagon-like peptide-1 receptor agonists (GLP-1RAs) are transforming medicine globally. Given the efficacy and the demand for these drugs for weight loss, significant and complex social implications will follow. Drawing on our current qualitative studies with users in Brazil, Denmark, Japan, the United States, and online communities, alongside our pre-GLP-1RA studies in ten additional countries, we identify nine emerging global trends. These include the profound sense of “normality” users describe after weight loss, demand driven by pervasive weight anxiety, and the willingness of many to endure significant side effects, costs, and sacrifices to maintain access. We also observe extensive medication tinkering, unregulated sourcing, changing dynamics in clinical consultations, entwinement with disordered eating, gendered patterns in use and outcomes, and the central role of social media in shaping beliefs and practices. Rather than reducing weight stigma, these drugs may intensify social judgments and inequalities. GLP-1RAs are thus not only biomedical innovations but also social technologies that reshape bodies, identities, and health systems. Anticipating their global impact requires integrating social science with health research and policy.

## Introduction

The accelerating uptake of new glucagon-like peptide-1 receptor agonists (GLP-1RA) is transforming weight management. Several factors predict massive global expansion of these “anti-obesity” drugs (like semaglutide and tirzepatide) in the years ahead. These include their efficacy; estimates of 2.1 billion adults clinically classified as overweight; state concerns over rising financial strain from “epidemics” of weight-related chronic disease; corporate profits to be made; current medical recommendation that they are “lifetime” drugs alongside evidence of rebound weight gain when discontinued; widespread international public awareness; and patient demand. When shifts in medicine happen at such a scale, and with such capacity to reshape both health and physical bodies, significant and complex social implications inevitably follow.

Identifying and anticipating these implications is our focus here. The authors represent four discrete teams of social scientists and allied biomedical researchers who have been studying the experience and social response to weight and weight loss for over three decades. Our work spans 14 countries in the Global North and Global South using diverse ethnographic methodologies focused on the lived experiences of those seeking to lose or otherwise control their weight (e.g., [1–4]). Our most recent rounds of data collection -- based in Brazil, Denmark, Japan, the US, and with online communities -- captured the introduction and early stages of acceleration of GLP-1RA use. In this essay, we identify nine globally convergent trends emerging from our early qualitative data as well as gray literature, different media sources, and peer-reviewed studies published to date, that constitute emergent evidence suggesting important social implications and complications arising from GLP-1RA use for the explicit purpose of weight management. We then contextualize these in terms of our pre-GLP-1RA research and suggest pressing questions for further research.

## Observation 1: The joy of feeling “normal”

From our prior work with those clinically classified with obesity at many different global sites, we would have predicted that any drug that accelerated and eased weight loss would be experienced by many as miraculous. It is approaching the “magic pill” that our and others’ pre-GLP-1RA studies identified as a deep, cross-cultural yearning [2,5]. In this context, a convergent theme emerging across our qualitative research programs is the extent to which many such users communicate happiness -- even ecstatic joy -- around these medications. They have given users what they have always wanted: to fit in and be “normal.” Users that lose substantial weight are acutely aware of the positive and often profound interpersonal and psychological changes that follow. It is liberating and rewarding to escape the judgment of others, but also of themselves. Like these two users in our US study: *“People just treat you differently...That’s just the way it is... it makes you feel good, you know? It just does”* and *“I* [used to] *just want to blend in and not stand out at all. And now, I don’t feel that... I feel normal.”*

And it is not just body size itself but also changes in appetite that are drawing closer to the imagined “normal” person that long-overweight users strive to be. The drug reveals the degree to which crippling, constant thinking about food had preoccupied their lives. For example, as one Danish user explained: *“It is so delightful when you are used to thinking about food and then getting free from these thoughts. Sometimes, I think it probably is how ordinary people feel”* [6].

From our prior research with bariatric patients, we can observe that the joy around lost weight tends to be uneven and sometimes transitory, and this is often profoundly and ironically shaped by the initially positive shifts people experience in their interactions with others [7]. It can build a deep, consuming fear around being judged even more harshly if they rebound. It can also be a double-edged emotional sword as people realize their better social treatment (promotions, romantic attention, positive comments, not being stared at) reveals how devalued they were when they were larger.

We currently have very limited understanding of the complex emotional navigation of GLP-1RAs by users once weight is lost, including those never clinically defined with obesity to begin with. For example: Facing a chronic challenge to prevent regain, how is mood -- and associated mental health -- shaped over the long-term? How does this then shape the ways GLP-1RAs are used through time?

## Observation 2. Weight anxiety, not just weight, shapes GLP-1RA demand

While most prescriptions for GLP-1RAs are for those who have long struggled with their weight, we also observe at all our field sites that the general pattern of uptake of GLP-1RAs for weight loss is not confined to those classified clinically with obesity based on the relevant diagnostic markers (chronic alterations in physiological function associated with high adiposity). Patients requesting GLP-1RA “off-label” prescriptions include those with “pre-clinical obesity” [8] (medically considered both “healthy” but “at risk,” such as defined by BMI or by definition as pre-diabetic). But it is sought by those who have no medical risk but are nonetheless “weight worried” [1], a potentially massive market increasingly targeted by telehealth companies [9]. It is no surprise. Our and others’ social science research has established that anxiety about weight is real, globalized, and observable at all body sizes [10]. This helps explain why surveys are identifying “looking and feeling better” as the major motivation for uptake for weight loss among both current and never-users (e.g., [11]).

Japan is a telling case in point. The nation has an extremely low obesity prevalence according to international standards of less than five percent, yet the drugs are being enthusiastically received. Based on a decade of fieldwork, we know that weight-related anxieties are common to adults of all sizes in Japan, and weight-shaming is frequent, explicit, and socially permitted. So, even though the threshold to access the medications for weight loss in Japan through insurance is steep (BMI > 35 kg/m2, plus an additional comorbidity), many Japanese people are electing to self-pay for GLP1-RAs sourced from online clinics.

In Brazil, the primary demographic utilizing these medications appears to consist of women who are white and of high socioeconomic status, even though the highest prevalences of obesity are observed among those of lowest socioeconomic status. Similarly, the press in Denmark has been questioning why GLP-1RA use is clustered in wealthier neighborhoods, where obesity prevalence is lowest. This suggests that economic and aesthetic concerns rather than weight determine the consumption of the medication, adding to social inequality in health.

Anxiety about weight in general and weight gain in particular motivates people across culturally diverse sites to seek consistent access to the GLP-1RAs. While weight anxiety is always present, we know that locally relevant holidays (e.g., Christmas, Hanukkah, New Year’s), seasons (e.g., summer), and events (e.g., weddings) can amplify weight anxiety [1]. How will this impact GLP-1RA supply and, especially, demand? For example, we can anticipate “seasonal use” -- such as for the “bikini-ready body” -- as a rising phenomenon, and one likely to pattern along social-economic lines like other costly cosmetic treatments.

## Observation 3: Willingness to suffer, sacrifice, and strategize

Another convergent theme across our work is the extent to which many users are willing to struggle and suffer to gain and maintain access to the drugs, extending well beyond what we have previously observed around people’s weight loss efforts or compared to other “elective” medications in an array of different settings. The ever-present social surveillance of bodies contributes to the shame and stigma that are drivers of the willingness to go to any lengths to achieve “thinness” [12–15]. There are several notable dimensions, including willingness to manage undesirable adverse effects (gastrointestinal symptoms, fatigue, dizziness), absorb high costs, and expend time and energy to navigate supply shortages [4].

Unpleasant gastrointestinal adverse effects of GLP-1RA drugs for weight loss are well documented in the medical literature but medically considered tolerable. Although the rate of discontinuation due to severe symptoms is low, the emerging evidence is that some potential users may never start in anticipation of them [16]. As might be anticipated, adverse effects emerge as a common point of discussion in our research interviews. But we are also seeing how others are willingly enduring sometimes extreme physical adverse effects when weight is clearly being lost [17]. Users are then also finding their own workaround strategies for managing. As one Brazilian shared: *“I’ve actually vomited so much that I fainted.... How did I stop vomiting? I lived off Coca* [Coke] *Zero... I would spend the whole day drinking Coca Zero, and I wouldn’t vomit”.* A Japanese woman cautions: *“*[T]*he side effects are severe. You have to put up with heartburn to see results”. “It’s so bad that you can’t eat. If you take the wrong dosage, you’ll vomit. It’s best to avoid activities that cause stimulation, such as drinking alcohol or going to the [Japanese] baths”.* Another woman in Japan explains how the very high motivation for weight loss is central to willingness: *“Other friends didn’t have as high a BMI as me, but they dropped out... because the side effects of nausea and drowsiness were too severe (they stopped using it)”.* One of our research participants in Denmark explained how she managed crippling post-injection headaches by calling in sick to work, rather than by missing or spacing injections [4]. In a clinical consultation specifically about the severity of side effects, another Danish woman patient nods to the doctor and responds, *“I don’t want to give up just because it’s tough.”* [4]

The other side of this is that patients are less willing to endure adverse effects if weight is not being lost. As a Danish user explained: *“I’ve reached a point where I’m saying, I’m not going to take it anymore. Because it’s not working... and I feel awful.”* But to highlight how complicated this is in practice, she then requested a higher dose at her next clinic visit.

Health insurance companies and national health systems are unwilling and unable to cover the full and considerable costs of these medications for “off-label” weight loss in the absence of diabetes or other serious comorbid diagnoses. While the self-pay price of medications varies markedly across countries, willingness to make substantial personal financial sacrifices to maintain non-covered costs is a notable and recurring cross-national theme. For many, the benefits of effective weight loss are considered priceless, or at least justifiable. In Brazil we were told: *“That’s something many people don’t realize. They see* [GLP-1RAs] *as expensive and think, ‘It’s absurd’. But if you do the math, you’re not spending those thousand reais on food anymore — you’re saving that money instead.”* And a US user says: “*I don’t care what it costs at this point. I wouldn’t have got the raise at work if I hadn’t lost the weight and had that extra confidence to ask…* [The drug] *has more than paid for itself.”* We heard the same argument many times. In Japan: *“If you calculate the cost performance, it is about 17,000 yen* [USD115] *per kilogram* [of weight lost.]*”* And life transitions are being defined by concerns over GLP-1RAs access. As a Danish user said: *“I’m currently living off my pension savings to make this work, but I’ll be retiring this summer, so I simply won’t be able to continue beyond that. As much as I want to, there’s a clear deadline for when I need to have lost the weight—it has to happen before then”.* [4] And in the US: “*If my insurance [through work] doesn’t cover the cost of medication, I am going to get another job offer so I can ask my boss to pay the difference. Or get a new job.”*

So, we need knowledge well beyond simply documenting adverse effects or calculating financial strain created by these drugs. For example, how are people managing the interaction between weight goals, weight loss, and side effects in their everyday lives? What creative patient-devised strategies for managing extreme adverse effects seem to work? What else is being sacrificed by users -- at what health, social, or economic cost -- to maintain access?

## Observation 4: Extensive and imaginative medication tinkering and unregulated seeking

Real-world cohort studies are noting that few patients follow the GLP-1RA titration schemes devised from premarket clinical trials [18]. In medical terms, such user decisions are usually considered a problem of non-adherence to treatment. But to social scientists, patients are never passive users of medical treatments and advice, and drugs themselves function as symbolic objects, carrying cultural, personal and social meanings that shape how people experience and interpret their use. Accordingly, non-adherence can also be recognized as one way people manage complex competing needs and goals and otherwise claim agency through decisions to accept or question medical authority (e.g., [19]). For a costly “lifetime” drug that contains the capacity to bestow the many societal benefits of a preferred body type, we would expect patients to be motivated to be doing exactly this.

Across all our field sites, we have observed users deploying a wide array of imaginative strategies for rationing expensive or scarce medication. Diabetes and related drugs are regularly readjusted by clinicians to improve efficacy and tolerability. But this “tinkering” is commonly patient led, operating without medical oversight. The most common are spacing out injections and stop-restart approaches reminiscent of yo-yo dieting. In Denmark, users explained how they “count clicks” on the injection pen to conserve part of a single dose. In Brazil, lower-income users described how they combine the latter with jumping directly to higher doses to avoid the cost of the recommended titration schedule. One explained how these practices are shaping drug distribution patterns, showing how lower-income individuals are unable to begin treatment with lower doses and gradually increase them in a stepwise manner: *“Here, we only have the higher dosag*e [in the pharmacies on the outskirts], *because if you don’t have the money to buy medication, you’ll probably start in the middle of the process* [a higher dose] *rather than from zero* [a minimal dose], *right?”* Prescription sharing - lending, borrowing and gifting medication with family, friends or acquaintances for whom it was not prescribed - is under-researched as a medical phenomenon [20], but can be common if medications are hard to access. We have encountered cases with GLP-1RA use, such as US spouses sharing a prescription when one has insurance cover (e.g., for diabetes) but the other does not (e.g., for weight loss).

The tinkering strategies shared in online communities are especially detailed and widely discussed and show how this is not only about managing cost. Reddit’s “MenOnThePen” posters discuss frustrations around effectiveness and suggest complex tinkering to accelerate weight loss. For example: *“If you’re super responding to the drug, cut back to half the dose for a week and see how that goes. Just do 30 clicks instead of the full 60. If you find it wearing off earlier, you can dose every four days to keep a lower more consistent level in your system.”* Online bodybuilding forums share information on “cycling” and “stacking” GLP-1RAs with other drugs to “bulk,” creating in the words of one such user a “killer combo” [21].

Medical professionals, we observe, find this tinkering both frustrating and concerning. Said one Danish nurse: *“It’s like* [patients] *don’t even see Wegovy as actual medicine,”* and continued to explain that it is treated like it’s just some kind of food you can take more or less of whenever you feel like it. “*It is medicine, damn it — it’s like they don’t respect that. If it were blood pressure meds, they wouldn’t be messing around like this,”* she added [4].

In interviews and analyses of online text, people are also sharing strategies for accessing GLP-1RAs through unregulated channels. This matches the limited medical research suggesting significant unauthorized and contraindicated use is a global phenomenon (e.g., [22] for the Middle East). Users are purchasing medication (which may be out of date or counterfeit) through their own social networks, at pop-up “weight loss” clinics, via local peer-to-peer online selling, or from international online vendors. For example, our research with online communities in Brazil has revealed a complex network operating on social media to commercialize human enhancement products, including steroids and GLP-1RAs, alongside other illegal items such as firearms and recreational drugs. In Denmark, Brazil, Japan, and the US alike, pharmacists have shared that they have never experienced anything like this, except - as some have also noted - around highly addictive pain relievers.

These observations raise unresolved questions: How can we ensure extensive patient tinkering is recognized in studies assessing drug efficacy and adverse effects? What are the best ways to disconnect GLP-1RAs -- as a serious medical intervention -- from being otherwise clumped with commercial weight loss products in the minds of users? How can we best track, understand, and address the real and profound patient motivation to access and use unregulated GLP-1RAs? Answers to all these will require significant and coordinated social science research.

## Observation 5: Changing dynamics in clinic consultations

Our and others’ pre-GLP-1RA research has detailed the highly fraught consultative process between patients medically classifiable with “obesity” and their primary care physicians. For example, patients detailed the frustrations of being treated as only a large body with any medical issues they report being turned back to their weight and avoiding consultations altogether if feeling judged for their weight. Doctors also avoided initiating conversations around weight, not only because explaining and managing “lifestyle” adjustments is complex and time-consuming, but also emotionally laden. GLP-1RAs are easing these tensions on both sides of clinic consultation, with an overall shift in favor of patient-stated preferences and goals. The content of conversations has also shifted, from diet and exercise to an almost-exclusive focus on GLP-1RAs. Through our ongoing work in Denmark -- where widespread access to GLP-1RAs happened earlier than other countries -- we have been able to confirm that patients are more willing to initiate discussions around weight loss, something which was previously rarely observed in the same primary care population [23].

While clinical encounters on “obesity” and GLP-1RAs treatment continue to be emotionally charged, patients are now also concerned with whether physicians will cooperate with their desire for the medication. Despite these challenges, most patients seem to achieve their goals. As some participants in Japan explained, simply raising the topic of weight gain to a healthcare professional may now result in the offer of an off-label prescription. This indicates a shift in terms of patient autonomy and power which aligns with what we are observing more generally: patients now arrive at consultations with information gleaned from extensive commercial marketing, social media, online discussion forums, and input from friends that is often inconsistent with standard medical advice offered by providers. It also resonates with surveys showing that only a small percentage of users identify healthcare professionals as their main information source on weight-loss drugs (e.g., 9% in one survey in the UK [11]).

Thus, the advent of GLP-1RAs as both a medical and social phenomenon presents a paradoxical situation regarding patient power, in which individuals may feel empowered by knowledge gained from non-medical sources, but simultaneously face new vulnerabilities and limitations. These complexities demand research attention. Are we seeing an overall gain in real “patient power”? Is elevated patient planning for, and engagement in, consultations around weight making GLP-1RAs safer or less safe, increasing or decreasing drug efficacy, or some combination of both? We also wonder how the (mostly negative) perception of the “obese patient” could be shifting for doctors, given this new evidence-based tool for controlling weight. That is, has obesity become more “instrumentalized” in ways that might reduce weight stigma in medical settings and its resulting harms?

## Observation 6: Complex entwinement with disordered eating

GLP-1RAs strongly suppress appetite and alter how users relate to food, generally reducing constant thoughts and interest in it. As a Danish user explained: “*Saxenda – that was effective! Oh wow, I actually developed an aversion to food. Just looking at, uh… I could be sitting there thinking, ‘Right, now I’m going to have a piece of chocolate,’ you know? And then I’d look at it and, ugh, I’d almost throw up. I just couldn’t handle it at all.”* Anecdotally, this was described as a silencing of “food noise,” a still loosely defined concept, but understood by users as a much-desired feature of GLP-1RAs rather than an adverse effect [17,24,25]. Hayashi et al. (2023) defined food noise as “heightened and/or persistent manifestations of food cue reactivity, often leading to food-related intrusive thoughts and maladaptive eating behaviors” [26, p3].

We do not deny that some individuals may struggle with these difficulties. However, the most compelling social aspect of “food noise” lies in its inseparability from the pathologization of hunger and eating, as well as from fatphobia. Hunger is a normal physiological response reframed as a problem to be solved by GLP-1RAs [27]. Food also plays a crucial social role in shaping individuals’ mental and social selves, yet it is increasingly treated as an issue to be managed pharmacologically. This is only possible due to structural fatphobia: in a fatphobic society, any thoughts about food that large-bodied individuals may have are stigmatized as inappropriate or undesirable [28].

This raises concerns about the complex entwinement between these drugs and disordered eating. In clinical and lay terms alike, GLP-1RAs–induced food avoidance is framed as a “good” outcome because it promotes weight loss. Thus, behaviors promoted for large-bodied individuals as “health-responsible” would be considered disordered in thinner ones. This creates a gray area for health professionals and users alike between what counts as “success” on GLP-1RAs and what would otherwise be deemed disordered eating. One U.S. user aptly described GLP-1RAs as “doctor-approved anorexia” [25]. This further complicates prevention strategies for eating disorders and obesity, intensifying contradictions in discourses on healthy eating, dietary restriction, and body norms [29].

As more users move to maintenance doses, serious questions emerge: Can we meaningfully distinguish between healthy and pathological use of GLP-1RAs to manage appetite? What constitutes a “healthy” GLP-1RA diet? What exactly is food noise, and how does it interact with food systems, diet culture, and fatphobia?

## Observation 7: “Sex” differences are likely gender differences

Clinical GLP-1RAs studies are identifying “sex” differences in reported GLP-1RAs adverse effects (women may have more and are more likely to discontinue) and weight loss outcomes (men may lose less). Pooled clinical studies are suggesting that the “hyper-response” of female-sexed biologies accounts for this (e.g., [30]). Yet, sex-specific mechanisms are unidentified [31]. From a social science perspective, medical studies often conflate sex and gender, such as assuming gender-differentiated data reflects a meaningful, underlying variation in sex biologies [32]. We should expect GLP-1RAs research to be no different.

We suggest a pressing need to situate GLP-1RAs clinical data in relation to what we consistently observe cross-culturally: women are both expected to conform to a narrower body norm and accrue greater social costs around deviations from them. For example, studies in the US suggest women are judged more harshly as “cheaters” for using GLP-1RAs drugs for weight loss than men [33]. But a more comprehensive view of gender and weight would suggest several additional elements to consider.

So, rather than beginning with an assumption of fundamental differences in sexed biologies, we should begin with the recognition that an array of as-yet-undocumented gendered differences in GLP-1RAs use patterns could explain many observed outcome differences for males and females. This could include willingness to use injectables or endure adverse effects for women due to more social costs to being labeled as overweight or combined use with steroids for men given a gendered norm for greater muscle bulk. In addition, for partnered people, the management of the use of GLP-1RAs (e.g., reminding, refilling) often falls to the person who manages the household. In Japan, this is women. Based on our research in Japan, the day-to-day use management may exhibit gendered differences which become embodied (e.g., are expressed in the biology of the users). Consistent with this, clinical studies report women have more medically accurate understandings of the drugs (e.g., [11]).

Thus, in efforts to understand “sex” differences in GLP-1RAs outcomes, we thus must first ask the question: How are user decisions that affect GLP-1RAs outcomes (e.g., medication tinkering and dietary strategies) explained through gender norms and expectations? It may be that many explanations for “sex” differences in GLP-1RAs outcomes are unneeded because they can be accounted for as gender differences.

## Observation 8: Social media matters

Commercial and internationalized marketing of GLP-1RAs drugs is currently on an upward trajectory as supply chains stabilize, supporting the general impression of GLP-1RAs as relatively safe and highly effective. Our shared observation is that the effects of this are nothing compared to the existing influence of social media. Studies machine-mining massive social media data sets, such as from Twitter/X, TikTok, and Reddit, reveal the incredible density, intensity, and global reach of this informal information- and belief-sharing around GLP-1RAs, and their penetration into wider non-medical discussions such as around reality TV, bodybuilding, and cosmetic effects (e.g., [34]). For example, the first 100 videos uploaded on TikTok under the hashtag #ozempic were viewed 70 million times [35]. Digging into these issues at our own field sites using qualitative research methods, we have detailed the many ways people on the ground are using social media to inform both their beliefs and decisions around GLP-1RAs use. This includes whether to begin or continue use, how to shape the outcome of conversations with prescribers, what adverse effects to expect, how to manage them, and where to (self-)source. As one user in Brazil explained: “*I only go on Facebook* [for how to use the drugs] *to clear up doubts. But it’s good... Like, seriously, instead of asking your doctor, you ask people in the group and see how many respond the same way to figure out if it’s right or wrong? Then later, it’s on the news.”* Of course, users also find that social media is a double-edged sword. As a user of *Chiebukuro* (a large Reddit-like platform heavily used in Japan) explained, *“Following a bunch of people... when I first started. I thought it would be helpful for navigating side effects and getting ideas for meals and snacks and stuff. But my Insta feed ended up feeling so toxic and was just making me feel yucky about it all.”*

From a social science perspective, these sorts of informal online interactions do much more than just share information, medically accurate and inaccurate. They can potentially create supportive communities and motivate collective action. They can affect psychological states and reshape people’s core sense of self. Given the intensity of interaction around GLP-1RAs, we need to ask: Is this creating greater “patient power”? Which forms of online engagement are beneficial or harmful and for whom, and what predicts the difference? How are existing social disadvantages being reduced or reproduced as people engage with each other in this rapidly evolving space?

## Observation 9: Weight-related stigma is unlikely to dissipate

Weight stigma is based in neoliberal ideologies of personal responsibility and belief that high body weights reflect moral failures, such as being lazy or lacking adequate self-control. It shares common roots with racism, grounded in the assumption that Black people are less capable of responsibly managing their bodily needs [36]. Our pre-GLP-1RAs research has collectively documented how pervasive, pernicious, and globalized it already was (e.g., [1]). There has been speculation that GLP-1RAs might reduce this stigma, in part because weight loss becomes easier and it helps reframe weight from a “personal failing” to a “medical condition” (e.g., [37]).

We are unconvinced that GLP-1RAs will herald a significant reduction in negative judgements around body weight. For one thing, GLP-1RAs use -- just as we have previously documented for bariatric surgery -- is consistently identified as “cheating,” “taking the easy way out,” and otherwise reflecting a lower moral value when compared to weight loss without medication. For example, in Japan qualification for a prescription requires evidence of an attempt at food restriction and exercise for 6 weeks. The underlying message is that pharmacological aids should be employed only when individuals “fail.” Another area in which we have observed a convergence of stigmatizing moral judgment has the argument that weight loss alone is not seen as adequate justification for priority access. A Brazilian user explained: “*So, most of the people I talk to, including my family and others... really question whether there’s a real cause—like if the person is truly obese and can’t control their eating, or if they have diabetes. Basically, if it’s genuinely a disease or a situation that’s out of control*.” A Japanese post on X (Twitter) also exemplifies this belief: *“There is no reason to use them if you don’t have obesity-related disease…* [they] *are being prescribed for anti-diabetes (which is fine) but* *for other reasons* [3 vomit emojis]*.”*

And, we have found in our prior research with people undergoing bariatric surgery, being “formerly fat” often retains a stigma even after extreme weight loss. Full escape is elusive. The broader social science around how stigma processes work more generally also shows how the distinctions between who is judged harshly as morally less-than and who suffers its social consequences are constantly reconstituted but nonetheless persistently track the same social and economic fault lines. That is, bodies that are maintained near a social ideal contain capital – increased capacities to elevate social, economic status and so on. Ideals will tend to shift to maintain benefits to those already with them.

The fact that GLP-1RAs remain both expensive and uncovered by many state health systems and private insurance companies also reinforces them as symbols of a Global North elite. Ultra-wealthy tennis star Serena Williams recently announced that that she is using GLP-1RAs to lose “extra mom weight” that persisted despite hours of exercise daily. She explained she was speaking out publicly to overcome the stigma of using the drugs for the goal of regaining body confidence [38]. Such perspectives will likely be amplified further as GLP-1RAs move into non-medical spaces, such as another tool in the fitness and wellness industry’s arsenal as part of the overall commercial “Uberfication” of weight loss that GLP-1RAs allow [17]. This sets a stage for “being fat” to become doubly stigmatized as a product of lower moral standing due to such factors as “laziness” *and* inability to pay.

Strings (2015) traced how obesity intersects with gender and race in the U.S. through the construction of “Black female sensualism”—the depiction of Black women as having insatiable appetites for food and sex, leading to behaviors deemed risky and resulting in “diseases” such as syphilis, tuberculosis, and, more recently, obesity [39]. While the former marked these women as potentially deadly to others, obesity redefined them as “social dead weight.” This notion legitimizes structural oppression by framing the supposed “burden” of social dead weight as a threat to family stability and public resources. The fact that women often have larger bodies and experience higher poverty levels intensifies this stigma, positioning them as both responsible for their “excess” and unable to afford market-based solutions like GLP-1RAs—thus portrayed as even more burdensome to the state. So, the pertinent question is not if stigma will be reduced by GLP-1RAs, but for whom it might be amplified and how it will intersect with race and class to ensure white dominance [40]. That is, will high body weight become even less socially acceptable in a world where a highly effective weight loss product is increasingly available to many, but not all?

## Final thoughts

When shifts happen in medicine at such a scale and with such capacity to reshape health and physical bodies, there are always significant social implications. Based on what we are observing in early research with GLP-1RA users in varied global locations, we have identified some emergent dimensions and related concerns. The complex dynamics between this new class of weight management drugs and the people using them defines a massive global project for the years ahead, and one that -- as we hope we have communicated -- social science is well-positioned to advance.
